# Direct Presentation Is Sufficient for an Efficient Anti-Viral CD8^+^ T Cell Response

**DOI:** 10.1371/journal.ppat.1000768

**Published:** 2010-02-12

**Authors:** Ren-Huan Xu, Sanda Remakus, Xueying Ma, Felicia Roscoe, Luis J. Sigal

**Affiliations:** Fox Chase Cancer Center, Philadelphia, Pennsylvania, United States of America; Oregon Health & Science University, United States of America

## Abstract

The extent to which direct- and cross-presentation (DP and CP) contribute to the priming of CD8^+^ T cell (T_CD8+_) responses to viruses is unclear mainly because of the difficulty in separating the two processes. Hence, while CP in the absence of DP has been clearly demonstrated, induction of an anti-viral T_CD8+_ response that excludes CP has never been purposely shown. Using vaccinia virus (VACV), which has been used as the vaccine to rid the world of smallpox and is proposed as a vector for many other vaccines, we show that DP is the main mechanism for the priming of an anti-viral T_CD8+_ response. These findings provide important insights to our understanding of how one of the most effective anti-viral vaccines induces immunity and should contribute to the development of novel vaccines.

## Author Summary

Professional antigen presenting cells fragment viral proteins and display some of the resulting peptides bound to MHC molecules at the cell surface. When virus-specific CD8^+^ T cells recognize these viral peptides they become activated, proliferate, and kill virus-infected cells to help rid the body of the virus. Two pathways have been described for the origin of the peptides presented by professional antigen presenting cells. In cross-presentation, the antigen presenting cells acquire the proteins from other cells which, in the case of a viral infection, must be infected. In direct presentation, the antigen presenting cells synthesize the proteins themselves and, therefore, during responses to viruses must be infected. However, the participation of direct presentation in anti-viral responses has never been deliberately demonstrated experimentally. In this paper we demonstrate that direct presentation occurs and is the main pathway to induce CD8^+^ T cells during infection with vaccinia virus. These findings provide important insights to our understanding of how one of the most effective anti-viral vaccines induces immunity and should contribute to the development of novel vaccines.

## Introduction

Activated CD8^+^ T lymphocytes (T_CD8+_) kill virus infected cells that display virus-derived peptides presented on cell surface MHC I molecules. Hence, T_CD8+_ play an essential role in the clearance of many primary viral infections. Moreover, the memory T_CD8+_ that remain after a primary infection or vaccination can also participate in resistance to disease following a secondary infection [1],[2],[3],[4]. While most cells of the body express MHC I and can therefore be targets of T_CD8+_ killing, their initial activation and expansion (priming) during many viral infections requires antigen presentation by bone marrow-derived (BMD) professional antigen presenting cells (APC) [5],[6],[7]. The two major routes of MHC I antigen presentation are direct- and cross-presentation (DP and CP). In DP the Ag presenting cell synthesizes the Ag. Thus, DP presentation requires the infection of the Ag presenting cell. In CP, uninfected cells acquire the Ags from other infected cells. While most cells can engage in DP, CP is a function of phagocytic BMD APC such as DC and Μφ [8],[9]. Several years ago we showed that when a virus cannot infect BMD APC, CP can still prime anti-viral T_CD8+_ [6]. Since then, the specific role of CP and DP in priming anti-viral T_CD8+_ has been a topic of discussion with some arguing that CP is in general important or essential, whereas others propose that it is physiologically irrelevant [8],[10],[11],[12],[13],[14]. The main reason for this ongoing discussion is a dearth of direct data supporting DP or CP as the main mechanism of T_CD8+_ priming in viral infections [15]. This most likely resulted from the difficulty in establishing appropriate experimental models that can exclude CP during an anti-viral response while maintaining similar levels of peptide-MHC complexes at the cell surface. For example, previous work by us and others has shown that (M)SIINFEKL expressed as a mini-gene during VACV infection is not a substrate for CP [16],[17] and further earlier work by Restifo et al. and Wherry et al. [18],[19] had shown that (M)SIINFEKL can prime T_CD8+_. Placing both pieces together, it could be argued that DP by VACV-infected cells has already been shown. However, because it does not require processing, VACV-(M)SIINFEKL infected cells express supra-physiologic K^b^-SIINFEKL complexes at the surface of infected cells (∼85,000 vs. 3,000 complexes per cell for VACV-full-length OVA [20]), has an extremely short half-life [21], and its ability to stimulate T_CD8+_ responses does not correlate with the very high levels MHC I-peptide complexes at the cell surface [19]. Furthermore, whether this construct requires BMD APC has not been investigated. Similarly, Norbury et al. has shown that SIINFEKL embedded in a rapidly degraded construct (Ub-R-NP-SIINFEKL-EGFP) is not cross-presented but induces a T_CD8+_ response [17]. However, while this construct requires processing, it is degraded very fast (10 minutes), resulting in faster K^b^-SIINFEKL formation and at least three times more K^b^-SIINFEKL complexes at the surface of infected cells as compared with a slowly degraded counterpart NP-SIINFEKL-EGFP [21]. Understanding how T_CD8+_ are primed, in particular for those viruses that are useful as vaccines, is of major importance as it may directly impinge on vaccine efficacy. Here, we explore the role of DP and CP in the priming of T_CD8+_ to vaccinia virus (VACV) which was used as the vaccine that eliminated smallpox and is proposed as a vaccine vector for a number of infectious diseases and cancer [22],[23].

## Results

### Direct presentation primes anti-VACV T_CD8+_

**Figure 1:**
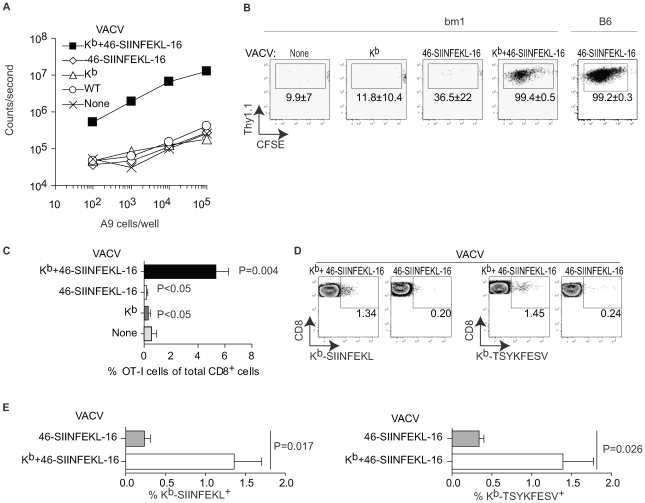
Direct presentation primes anti-VACV T_CD8+_. **A**) A9 cells (H-2^k^) were infected with the indicated viruses (10 PFU/cell) for 2 h and Ag presentation determined using the K^b^-SIINFEKL specific hybridoma B3Z as previously reported [42]. Data correspond to the average of two wells and is representative of two similar experiments. **B**) bm1 or B6 mice were adoptively transferred with 10^6^ CFSE labeled OT-I cells and infected IP with 10^6^ PFU of the indicated viruses. OT-I proliferation in the spleen was determined by FACS on day 4 PI. Plots correspond to a pool of three mice and are representative of three experiments. The numbers in the plots are the average ± SEM for the three experiments. **C**) As in **B** for bm1 mice but displayed as the % of OT-I cells of the total CD8^+^ cells in the host. The P values shown are against the uninfected host. **D**) bm1 mice were infected IP with 10^6^ PFU of the indicated viruses. Seven days later the SIINFEKL and TSYKFESV-specific T_CD8+_ were determined in the peritoneal wash by staining with the indicated K^b^ tetramers. Each plot corresponds to a pool of three mice from a representative experiment of three. Data are gated on CD8+ cells. **E**) Summary data for the three experiments in **D**. P value determined by one-tailed T test.

Previous work by others showing clustering of TCR transgenic TCD8+ (T_CD8+_) with VACV infected APC suggested that DP can prime anti-VACV T_CD8+_ responses [24],[25]. However, this work did not formally prove that this clustering resulted in effective priming or T_CD8+_ expansion. To directly look into this issue, we made VACV-K^b^+46-SIINFEKL-16, a double recombinant VACV co-expressing the MHC I molecule H-2 K^b^ and 46-SIINFEKL-16, a truncated form of chicken ovalbumin (OVA, 386 amino acids) comprising the K^b^-restricted immunodominant determinant SIINFEKL preceded by 46 and followed by 16 amino acids from the natural OVA sequence. Of interest, this construct is a substrate for DP but not CP [26]. As shown in Figure 1A, K^b^-negative A9 cells infected with VACV-K^b^+46-SIINFEKL-16 induced B3Z T cells, a T cell hybridoma that produces β-galactosidase (β-gal) upon recognition of K^b^-SIINFEKL and can be used to compare amounts of K^b^-SIINFEKL at the surface of cells [27],[28]. On the other hand, control A9 cells infected with VACV wild type (VACV-WT) or the single recombinants VACV-K^b^ and VACV-46-SIINFEKL-16, did not induce β-gal in B3Z cells. Additional controls showed that B3Z cells were induced when infecting Kb^+^ MC57G fibrosarcoma cells with VACV-K^b^+46-SIINFEKL-16 or VACV-46-SIINFEKL-16 but not with VACV WT or VACV-K^b^ (not shown). Thus, virus encoded K^b^ can directly present virus encoded SIINFEKL in tissue culture. To determine whether virus-encoded K^b^ results in DP *in vivo*, B6.C-H2^bm1^/ByJ mice [bm1 mice; a C57BL/6 (B6) congenic strain carrying a mutant K^b^ allele (K^bm1^)] were adoptively transferred with CFSE labeled splenocytes from OT-I TCR transgenic mice [29] and infected with various viruses. As shown in Figure 1B and C, the OT-I T_CD8+_ proliferated extensively and significantly increased in proportion relative to the endogenous T_CD8+_ population when the mice were infected with VACV-K^b^+46-SIINFEKL-16 but not when infected with VACV-K^b^ or VACV-46-SIINFEKL-16. Some loss of CFSE fluorescence in a sizeable number of the OT-I cells in mice infected with VACV-46-SIINFEKL-16 (Figure 1B, center panel) was not reproducible (see the wide SD), and was most likely background because the proportion of OT-I cells did not increase significantly in these mice (Figure 1C). Similar results were obtained in the D-LN of mice inoculated IP and in the spleen and D-LN of mice inoculated SC (Figure S1A). In control experiments, VACV-46-SIINFEK-16 strongly stimulated OT-I cells in B6 mice (Figure 1B, right panel). Of note, the OT-I cells in B6 mice infected with VACV-46-SIINFEKL-16 expanded much more than in bm1 mice infected with VACV-K^b^+46-SIINFEKL-16 as indicated by their significantly higher proportional increase (to 42.6±6.3% of total T_CD8+_, not shown) most likely indicating that K^b^ expressed by the virus cannot faithfully reproduce endogenous K^b^ expression. Regardless, because OT-I T_CD8+_ cells recognize SIINFEKL in the context of K^b^ but not of K^bm1^ [30],[31],[32], the results with bm1 mice strongly suggest that infected cells can directly present antigen to OT-I cells *in vivo*.

While OT-I cells have been used extensively to detect antigen presentation *in vivo*, there is the caveat that, because of their high TCR affinity, their priming requirements likely differ from those of a polyclonal naïve repertoire. In fact, their value as a tool in priming and T cell kinetics experiments has been questioned [33]. Thus, to extend our findings to a polyclonal naïve repertoire we determined whether infection with a K^b^-expressing virus can induced an endogenous T_CD8+_ response in bm1 mice. For this purpose, we infected bm1 mice with 10^6^ PFU VACV-46-SIINFEKL-16 or VACV-K^b^+46-SIINFEKL-16 and, seven days PI, we determined the endogenous T_CD8+_ responses to SIINFEKL and also to the dominant K^b^-restricted genuine VACV determinant TSYKFESV [34] using appropriate K^b^ tetramers. We found that VACV-K^b^+46-SIINFEKL-16 but not VACV-46-SIINFEKL-16 was able to stimulate significant anti-K^b^-SIINFEKL and anti-K^b^-TSYKFESV responses in the peritoneal cavity of bm1 mice (Figure 1D and E) demonstrating that VACV infected cells can use DP to expand polyclonal (non-transgenic) T_CD8+_ to the recombinant determinant SIINFEKL and also to TSYKFESV in bm1 congenic mice. Similar results where obtained for the spleens, peritoneal washes and lymph nodes of mice infected with 10^5^ PFU of the viruses either IP or SC (Figure S1B, showing examples of two individual mice to demonstrate reproducibility). The response was K^b^-peptide and not K^b^-allo -specific because K^b^-SIINFEKL tetramers stained a significant proportion of T_CD8+_ in bm1 mice infected with VACV-K^b^+ 46-SIINFEKL-16 but not with VAC-K^b^ (Figure S1C and D). The data also indicate that the repertoire of bm1 mice includes at least some TCRs capable of recognizing TSYKFESV and SIINFEKL in the context of K^b^. However, the response in bm1 mice, in particular against TSYKFESV, was much smaller than in B6 mice (see, for example, Figure 3D and E). This may be due to different expression of virus-encoded vs. endogenous K^b^ (as with the OT-I cells) and/or defective positive selection of K^b^-restricted T cells in the bm1 thymus as previously reported by Nikolic-Zugic et al. [32]. The fact that we detected K^b^-SIINFEKL specific cells in bm1 mice while Nikolic-Zugic did not may be because we used a more potent antigenic stimulus (OVA encoded by VACV vs. OVA-loaded cells) and/or that they detected the responses using the 51Cr release assays while we used tetramer staining.

### Bone marrow derived cells are responsible for the direct priming of anti-VACV T_CD8+_

**Figure 2:**
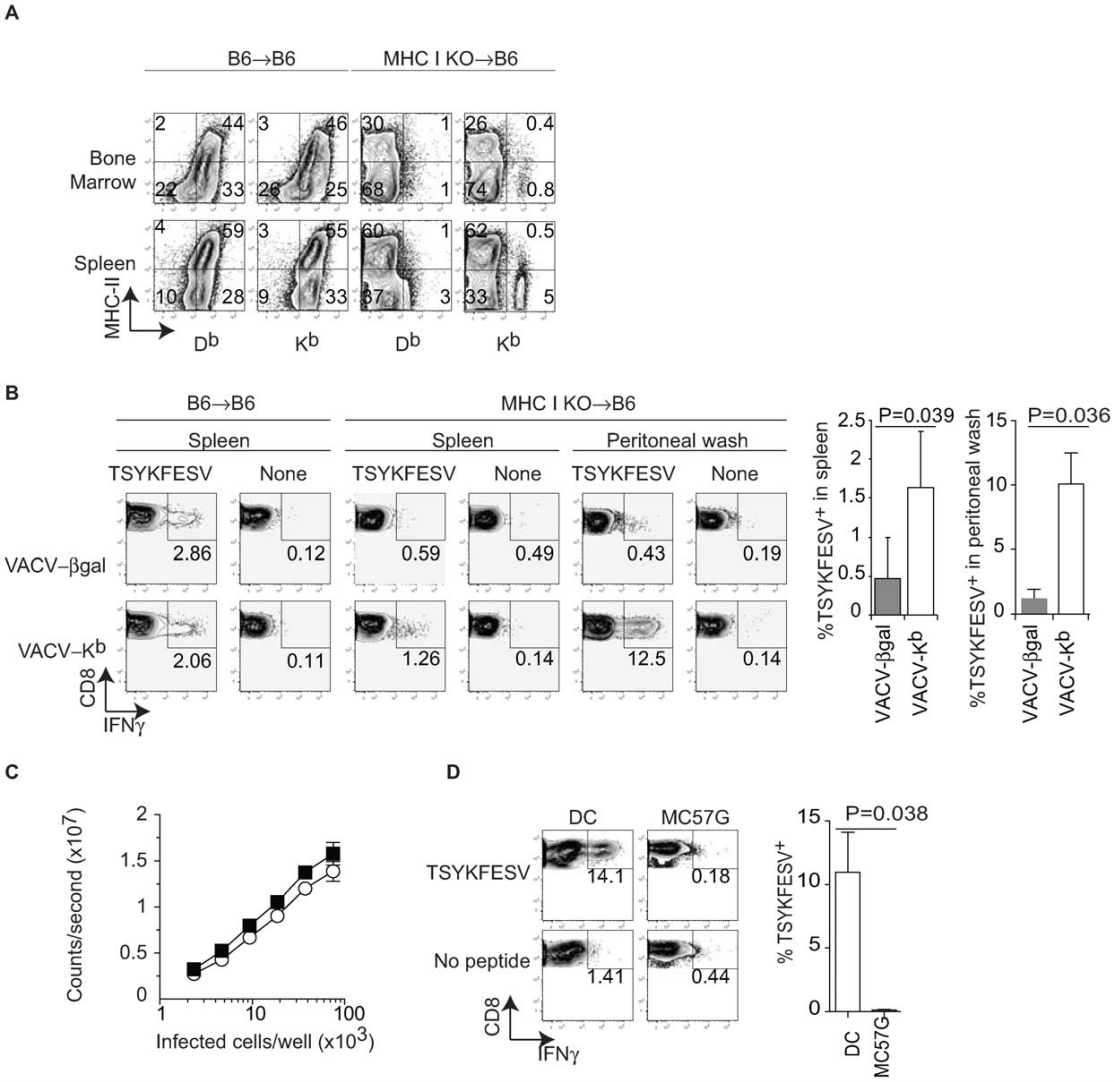
Only bone marrow derived cells prime anti-VACV T_CD8+_ by DP. **A**) B6 mice were lethally irradiated (400+500 rads) and reconstituted with bone marrow from MHC I KO mice. Expression for MHC II and MHC I was determined in the indicated organs four months later. Data correspond to one mouse and is representative of three mice/group and three similar experiments. **B**) The indicated bone marrow chimeric mice were infected IP with 10^6^ PFU of the indicated recombinant VACV. Seven days PI TSYKFESV-specific responses was determined in the spleen and peritoneal wash following restimulation with DC2.4 cells that had been pulsed or not with TSYKFESV. FACS plots correspond to a pool of three mice and are representative of two similar experiments. Data are gated on CD8^+^ cells. The column graphs on the right are the summary data for the spleen and peritoneal wash of the two experiments. The P values are for one-tailed T test. **C**) BMD DC and MC57G cells were infected for 2 h with 1 PFU/cell VACV 46-SIINFEKL-16, serially diluted as indicated and Ag presentation determined using the K^b^-SIINFEKL specific hybridoma B3Z as previously reported [42]. Data correspond to the average of three wells and is representative of two similar experiments. There was no statistical significance for the small difference observed between the two curves (P = 0.6323 in two-tailed T test). **D**) MHC I KO →B6 bone marrow chimeric mice were inoculated IP with 10^6^ of the indicated cells that had been infected with 10 PFU/cell WT VACV. Seven days later the TSYKFESV-specific response was determined in the peritoneal wash (following restimulation with DC2.4 cells that had been pulsed or not with TSYKFESV). FACS plots correspond to a pool of three mice and are representative of two similar experiments. Data are gated on CD8^+^ cells. The column graph on the right summarizes the two experiments. The P value is for a one-tailed T test.

The previous data strongly suggested that DP can prime anti-VACV T_CD8+_. However, the experimental system had the caveat of using a semi-allogeneic system and that it does not distinguish between direct priming by infected cells of bone marrow vs. non-bone marrow (parenchymal) origin. We have previously used bone marrow chimeras with deficient expression of MHC I at the cell surface of BMD cells (from TAP1 deficient mice) to show that only BMD APC can prime T_CD8+_ responses to VACV and other viruses [6]. Thus, to directly address the role of DP by BMD APC in the priming of endogenous T_CD8+_ responses, we reconstituted lethally irradiated B6 mice with bone marrow from mice deficient in H-2 K^b^ and D^b^ (MHC I KO). Four months after reconstitution, most cells in the spleen, bone marrow (Figure 2A), and peritoneal wash (not shown) of MHC I KO→B6 mice lacked K^b^ and D^b^ expression with the exception of a small residual population of cells in the bone marrow (∼2%) and spleen (∼5%), most of which were not “professional” APC because they lacked MHC II expression. Thus, the vast majority of professional APCs in aged MHC I KO →B6 mice lack DP as well as CP abilities due to deficient MHC I expression. However, because these APC lack MHC I but otherwise their antigen presentation machinery is intact, they could regain at least some DP capabilities if infected with a K^b^-expressing virus. In addition, the APC in MHC I KO → B6 mice should also be capable of presenting pre-formed K^b^-peptide complexes obtained thorugh membrane exchange (ME) with parenchymal cells, a mechanism of antigen presentation that was discovered somewhat recently [35],[36],[37],[38],[39]. Four months after reconstitution, the MHC I KO→B6 and B6→B6 control mice were infected with recombinant VACV-β-gal or with VACV-K^b^. Seven days later, the anti-TSYKFESV T_CD8+_ response was measured in different organs by restimulating lymphocytes for 4 h with APC pulsed with TSYKFESV in the presence of brefeldin A followed by surface (CD8) and intracellular IFN-γ staining (IIS) and FACS analysis. We found that VACV-β-gal infection of MHC I KO→B6 mice resulted in an anti-TSYKFESV response in the spleen that was very reduced as compared to B6→B6 controls (Figure 2B), confirming our previous work [6] demonstrating that BMD APC are essential for the anti-VACV T_CD8+_. Moreover, this experiment shows that priming by ME (which was unknown at the time of our previous work) from parenchymal cells to APC does not play a dominant role in the anti-VACV T_CD8+_ response. More important, we found that much of the anti-TSYKFESV response was significantly restored when the MHC I KO→B6 chimeras were infected with VACV-K^b^. Furthermore, MHC I KO→B6 mice mounted a significantly stronger response to TSYKFESV in the peritoneal wash when infected with VACV-K^b^ as compared with VACV-β-gal (Figure 2B). This strongly supports the hypothesis that BMD cells, but not parenchymal cells, infected with VACV can use DP to prime an endogenous polyclonal T_CD8+_ response to the VACV immunodominant determinant TSYKFESV.

To determine differences in DP by BMD APC vs parenchymal cells, we infected BMD DC (as a model for APC) and MC57G cells (as a model for parenchymal cells) with VACV 46-SIINFEKL-16 and measured the relative amount of K^b^-SIINFEKL complex at the cell surface using B3Z cells (which do not require BMD APC for stimulation). We found that the two cell types were quantitatively comparable in their ability to stimulate B3Z cells indicating that they expressed roughly similar amounts of K^b^-peptide complexes at the cell surface (Figure 2C). However, while infected WT DC primed an anti-VACV response *in vivo*, MC57G cells did not (Figure 2D). This strongly suggests that the difference in the ability of BMD APC vs parenchymal cells to prime T_CD8+_ to an Ag that needs processing is qualitative rather than quantitative and further suggested that DP or ME by parenchymal cells do not play a major role in the anti-VACV T_CD8+_ response. Of note, the priming following inoculation of infected DC was due to their expression of MHC and not to an adjuvant effect because infected DC deficient in MHC I did not induce an anti-VACV response (not shown).

### CP is dispensable for an efficient T_CD8+_ response to VACV

**Figure 3:**
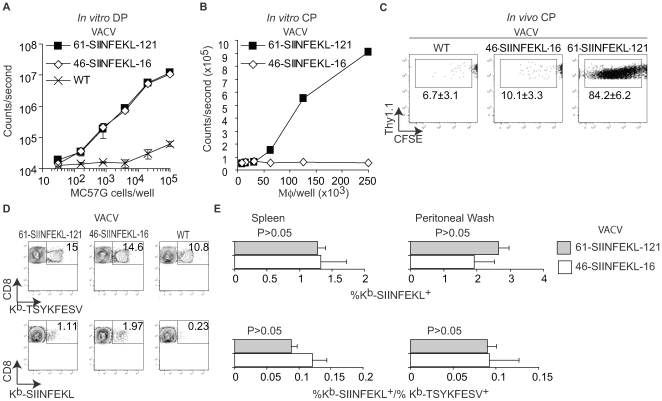
CP is not essential for efficient anti-VACV T_CD8+_ responses. **A**) Direct presentation *in vitro*: MC57G cells (H-2^b^) were infected with the indicated viruses (10 PFU/cell) for 2 h and Ag presentation was determined using B3Z cells as in Figure 1. Data correspond to the average of two wells and is representative of two similar experiments. **B**) Cross presentation *in vitro*: A9 cell were infected with the indicated viruses (1 PFU/cell) overnight UV irradiated for 2 h on ice, and fixed with paraformaldehyde followed by incubation with 1×10^6^ BM-derived B6 macrophages for 1 h. CP was determined using B3Z cells. **C**) 5×10^6^ CFSE labeled OT-I cells were transferred to B6 mice. One day later, the mice were injected 5×10^6^ virus-infected UV treated and paraformaldehyde fixed A9 cells (as described in **B**) subcutaneously. OT-I cell proliferation was determined by FACS in spleen on day 4 PI. The data in the plot corresponds to a pool of three mice and is representative of three experiments. Numbers are the mean ± SD for the three experiments. The differences between WT and 46-SIINFEKL-16 were not significant. The differences between 61-SIINFEKL-121 and WT had P<0.00003 by one-tailed T test. Data are gated on CD8+ and Thy 1.1 + cells. **D**) C57BL/6 mice were infected with 1×10^6^ PFU of the indicated viruses IP. Seven days PI the SIINFEKL- and TSYKFESV-specific T_CD8+_ was determined in the spleen and peritoneal wash by staining with the indicated K^b^ tetramers. Data correspond to a representative mouse of three and is representative of three experiments. Data is gated on CD8^+^ cells. **E**) Quantification of **D**. Upper panels shows the percentage of K^b^-SIINFEKL specific T_CD8+_ of total CD8+ cells in spleen and peritoneal wash, and lower panel shows the data normalized as (%K^b^-SIINFEKL^+^ T_CD8+_/% K^b^-TSYKFESV ^+^ T_CD8+_). The data represents means ± SD of three mice. There was not statistically significant differences by T test analysis between the mice infected with 61-SIINFEKL-121 vs. 46-SIINFEKL-16.

The data thus far demonstrated that anti-VACV T_CD8+_ responses can be induced by DP. However, the experiments did not address to what extent DP and CP contribute to priming during VACV infection. Using a transfection/infection model, we have recently shown that during VACV infection, 61-SIINFEKL-121 (a truncated form of OVA comprising SIINFEKL preceded by 61 and followed by 121 AA of the natural OVA sequence) and 46-SIINFEKL-16 are processed for DP with similar efficiency. However, even though both constructs have extended half-lives, only 61-SIINFEKL-121 is processed for CP [26]. Hence, we tested whether the antigenic properties of 61-SIINFEKL-121 and 46-SIINFEKL-16 were maintained when expressed in recombinant viruses. As expected, MC57G cells infected with either virus induced B3Z cells by DP with identical efficiency (Figure 3A). On the other hand, consistent with our results with the transfection/infection system [26], infection of A9 cells with recombinant VACV 61-SIINFEKL-121 but not with VACV 46-SIINFEKL-16 resulted in CP *in vitro* (Figure 3B) and CP to OT-I cells *in vivo* (Figure 3C). Next, we infected mice with 10^6^ PFU of VACV 61-SIINFEKL-121 or VACV 46-SIINFEKL-16 IP and, using specific MHC tetramers, we determined the potency of the anti-SIINFEKL T_CD8+_ response in the peritoneal cavity and in the spleen. The anti-TSYKFESV response served as an internal control and to normalize the anti-SIINFEKL response. We did not find any significant difference between the two viruses (Figure 3D and 3E). Similar results were obtained for mice infected SC and/or with higher or lower viral doses (10^8^ PFU and 10^4^ PFU) determined by either tetramer staining or IIS (not shown). Because 46-SIINFEKL-16 is not cross-presented, these results imply that CP is dispensable for the induction of a maximal T_CD8+_ response during VACV infection independent of the dose or route.

**Figure 4:**
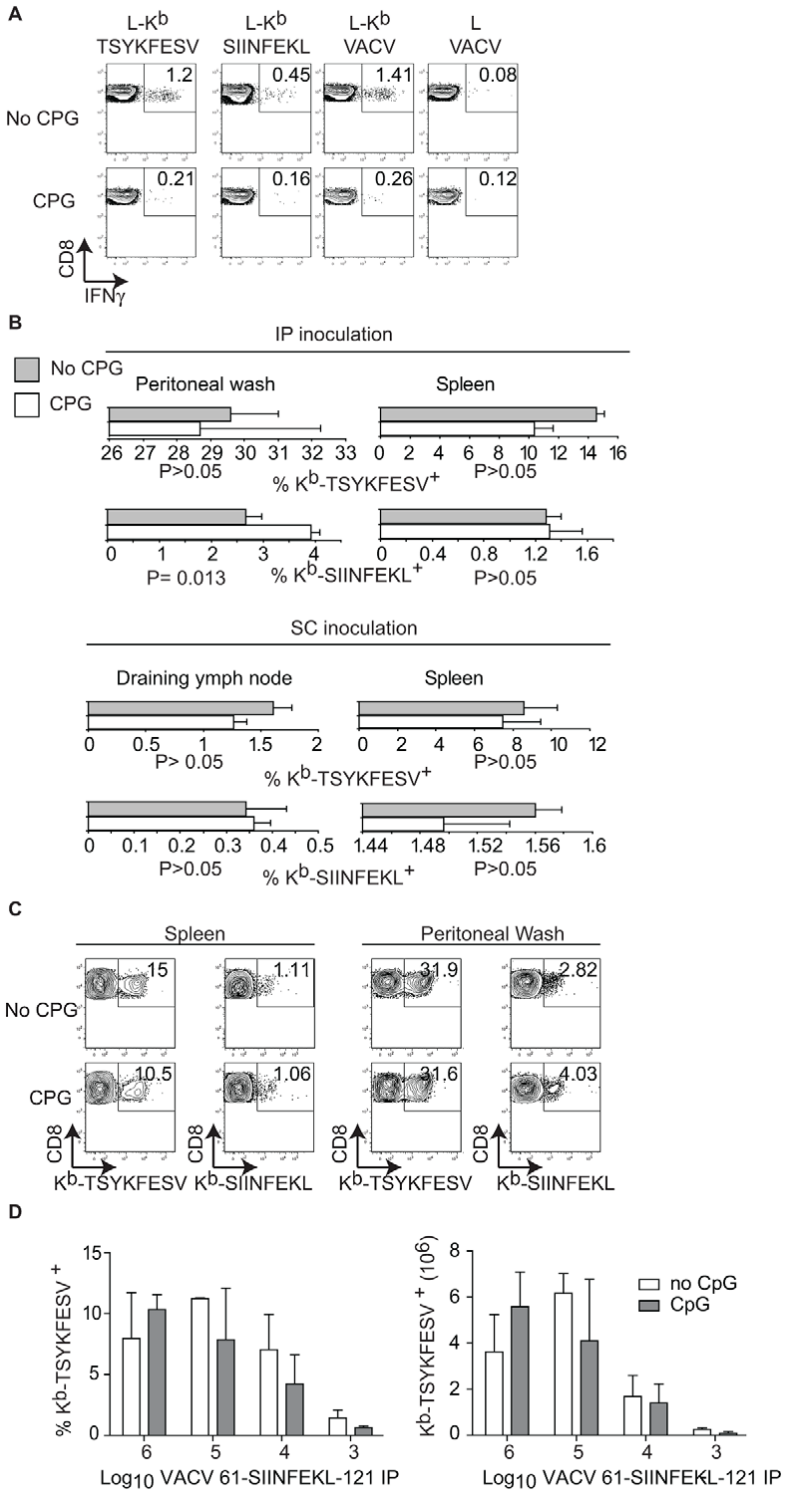
TLR-ligands do not block the T_CD8+_ response to live VACV. **A**) A9 cells infected with VACV 61-SIINFEKL-121 UV treated and fixed with 2% paraformaldehyde were inoculated to mice previously inoculated i.v. with PBS (mock, upper panels) or CpG (lower panels). Seven days PI the splenocytes of these mice were restimulated for 4 h *in vitro* with L cells transfected with K^b^ (L-K^b^) or control K^b^-negative L cells (L cells) that had been either pulsed with SIINFEKL or TSYKFESV; or infected with VACV. Following restimulation, the cells were stained as indicated and analyzed by FACS. Data represent a pool of three mice. Data is gated on CD8^+^ cells. **B**) Quantification of K^b^-SIINFEKL and K^b^–TSYKFESV tetramer+ cells in the spleen and peritoneal wash (IP inoculation) or spleen and D-LN (SC inoculation) in mice that had been inoculated i.v. with PBS (gray bars) or CPG (white bars) and infected with 10^6^ PFU of VACV 61-SIINFEKL-121 IP. Data are the average ± SEM of three mice and representative of three similar experiments. P values by two-tailed T test. **C**) Representative examples from individual mice in B inoculated IP. Data are gated on CD8^+^ cells. **D**) Relative (left, as a percent of total T_CD8+_) and absolute (right) numbers of K^b^–TSYKFESV tetramer^+^ cells in spleen of mice inoculated IP with the indicated doses of VACV 61-SIINFEKL-121 stained 7 days PI. Data correspond to three mice/group and are from an experiment different to that in **B** and **C**. No statistically significant differences were found by two-tailed T test analysis comparing CpG treated and non-treated mice with any of the virus doses.

It has been shown that pre-treatment of mice with the TLR9 ligand CpG induces maturation of DC, blocks CP, and inhibits the T_CD8+_ response to herpes simplex virus (HSV) and influenza virus *in vivo* [11]. In our hands, this treatment also inhibited CP because mice treated with CpG had significantly reduced T_CD8+_ responses to SIINFEKL and TSYKFESV when inoculated IP with L cells that had been infected with VACV 61-SIINFEKL-121 to induce Ag expression, and then treated with UV light and paraformaldehyde to eliminate any traces of live virus (Figure 4A). However, CpG treatment did not significantly reduce priming of anti-SIINFEKL or anti-TSYKFESV T_CD8+_ in mice that had been infected with 10^3^–10^6^ PFU VACV 61-SIINFEKL-121 (Figure 4B–D) or VACV 46-SIINFEKL-16 (not shown), even though the potency of priming decreased with reduced virus dose. These results further imply that CP is dispensable for the induction of efficient anti-VACV T_CD8+_ responses following infection with live VACV. In fact, the only significant change that we observed with CpG treatment was an increase in the anti-TSYKFESV response in mice inoculated with 10^6^ PFU. The reason for this increase is unknown but we speculate it may be due to an adjuvant effect of CpG. Why this increase was not observed for other viral doses or for SIINFEKL remains to be explored.

## Discussion

We have previously shown the strict requirement for BMD APC in the priming of T_CD8+_ responses to VACV and other viruses and that CP can prime T_CD8+_ when DP by BMD APC is abrogated [6],[7]. However, the extent whereby the DP and CP pathways contribute to an anti-viral response when both mechanisms are possible remained elusive because of the difficulty in ablating CP. Hence, priming of an anti-viral response exclusively by DP has never been demonstrated intentionally. In this paper we developed novel methods to disrupt CP and used them to demonstrate efficient priming of anti-VACV T_CD8+_ by DP following IP and SC inoculation. Furthermore, we show that when DP is available, CP is dispensable for eliciting a maximal anti-VACV T_CD8+_ response.

It has previously been shown that some anti-viral T_CD8+_ responses require or are partially dependent on CP. For instance, Shen et al. showed a decreased T_CD8+_ response to influenza virus in the absence of Cathepsin S, which is required for the processing of exogenous Ag via the TAP independent pathway [40] while Wilson et al.[11] showed that inhibiting CP by administration of the TLR9 ligand inhibited the T_CD8+_ response to HSV 1. In the case of VACV, we and others have shown that VACV encoded Ags can indeed be cross-presented [16],[41],[42]. Attempts have also been made to quantify the contribution of CP and DP to the overall anti-VACV response. For instance, Gasteiger et al. has shown that the T_CD8+_ response to the MVA strain of VACV requires CP [43]. However, the different requirements for this strain for VACV could be due to the fact that MVA is highly deficient in viral replication. Also, Basta et al. and Shen et al. [44],[45] compared the T_CD8+_ responses to recombinant VACV expressing US2 and/or US11 from human cytomegalovirus (HCMV) US11, or β-gal as a control. Because these viruses induced T_CD8+_ responses to different degree depending on the route of infection, it was concluded that CP and DP contribute differentially to the anti-VACV T_CD8+_ response. However, the conclusions assumed that US2 and US11 shut down DP *in vivo*, which has never been demonstrated. Moreover, the conclusions were based on the presumption that molecules that inhibit the MHC I pathway could not maintain functionality and block CP when transferred from the Ag donor cell to the APC. However, more recent work from the Cresswell laboratory [46] showed that exogenous ICP47 from HSV (another protein that blocks MHC I Ag presentation) can block CP making the supposition doubtful. In addition, while the direct interaction between infected APCs and TCR transgenic cells specific for a virus encoded Ag has been shown [24],[25], a clear demonstration of direct priming of naïve polyclonal anti-viral T_CD8+_ by infected APC expressing MHC I-peptide at relatively normal levels was still lacking. Here we have used four novel models to demonstrate that *in vivo* priming of anti-viral T_CD8+_ by DP occurs and that CP is dispensable to efficiently prime anti VACV T_CD8+_ *in vivo*. First, we used a semi-allogeneic model where the restricting MHC I and the Ag were exclusively encoded by VACV. Using this model we showed that following SC or IP infection, DP can stimulate TCR transgenic OT-I T cells and can also prime endogenous polyclonal responses to a recombinant (SIINFEKL) and an authentic (TSYKFESV) VACV determinant. It should be pointed out, however, that the OT-I responses in bm1 mice were not as strong as in B6 mice probably because the expression of endogenous MHC I cannot be faithfully replicated by virus-driven expression and, in the case of the endogenous responses, the repertoire capable of recognizing peptides in the context of K^b^ may be reduced in bm1 mice. Second, using bone marrow chimeras that lack expression of MHC I on BMD APC and infecting with VACV-K^b^ or control virus or inoculating with infected cells of bone marrow or parenchymal origin, we also showed priming by DP against TSYKFESV following IP or SC infection or DC inoculation. Further, we ruled out the transfer of preformed peptide MHC I complexes [35],[36],[37],[38],[39] from endogenous or inoculated parenchymal cells as a major mechanism for priming during VACV infection. In addition, these data also confirmed our earlier work that the priming of anti-VACV T_CD8+_ requires Ag presentation by BMDC [6]. Third, by comparing T_CD8+_ responses to 46-SIINFEKL-16, a form of OVA that is not cross-presented and 61-SIINFEKL-121, a form of OVA that is cross-presented [26], we showed that CP is not essential for full-fledged T_CD8+_ responses to VACV independent of the route or dose of infection. Fourth, we showed that *in vivo* blockade of CP using the TLR9 ligand CpG does not inhibit the anti-VACV T_CD8+_ response as it did for HSV [11]. Together, our experiments demonstrate that DP is the main mechanism for the priming of anti-VACV T_CD8+_.

Current models of Ag presentation mostly based on inert Ag suggest that APC acquire Ag in tissues, then mature, and finally migrate to the draining lymph node (D-LN) to prime T cells. While it is straightforward to imagine an uninfected APC loaded with Ag migrating to the D-LN, it is also possible to imagine that an APC infected with a cytopathic virus such as VACV would be migration-impaired. Thus, a remaining important question is to determine whether infected APC are still able to migrate to the D-LN following SC inoculation. Alternatively, free viral particles could reach the D-LN through afferent lymphatic capillaries as was shown with large inoculums of vesicular stomatitis virus [47] infecting D-LN resident APC. The site of priming following IP infection is more obscure and while it is possible that it occurs in the (para-thymic) D-LN, it is tempting to speculate that the peritoneal cavity, which has large nuber of BMD Μφ, could act as a secondary lymphoid organ.

In summary our work demonstrates that DP is the main mechanism responsible for the priming of anti-VACV T_CD8+_ responses. These results are important for our general understanding of anti-viral T_CD8+_ immunity and for the use of VACV as a vaccine vector.

## Materials and Methods

All experiments involving mice were performed according to Fox Chase Cancer Center guidelines for the care and use of laboratory animals and all animal studies were approved by the Fox Chase Cancer Center Institutional Animal Care and Use Committee.

### Cells and viruses

All cells were grown at 37°C in an atmosphere of 5% CO2 in RPMI 1640 medium supplemented with 10% FCS, 2 mM L-glutamine, penicillin-streptomycin, 0.01 M HEPES buffer and 5×10^−5^ M 2-ME (Sigma-Aldrich, St. Louis, MO). As L cells (H-2K) we used its derivative A9 (ATCC no. CCL-1.4). L cells stably expressing K^b^ (L-K^b^) [34], were a gift from Drs. Yewdell and Bennink. MC57G cells (ATCC no. CRL-2295) are a C57BL/6 fibrosarcoma (H-2^b^). B3Z is a CD8 T cell hybridoma that produces β-gal upon recognition of SIINFEKL in the context of the H-2K^b^ molecule [48] without the need of costimulation. Hela S3 (CCL –2.2) and BS-C-1 (CCL-26) were used to propagate virus and determine VACV titer. *In vitro* differentiation of DC and Μφ from bone marrow was as previously described [42].

VACV stocks were prepared as described [49] VACV-46-SIINFEKL-16 and VACV-61-SIINFEKL-121 were previously described [26]. The VACV-K^b^ in Figure 2 was a gift from Drs. Jonathan Yewdell and Jack Bennink (NIH, Bethesda, Maryland) and co-expresses β-gal and K^b^ disrupting the TK gene. VACV-β-gal was generated by homologous recombination into the TK gene using the plasmid pSC65 as described [50]. The VACV-k^b^ in Figure 1, VACV-Kb+46-SIINFEKL-16 and VACV 61-SIINFEKL-121 were generated by homologous recombination using appropriate constructs inserted in the plasmid pRB21 and selection of large plaques as described [50]. The correct sequence of the recombinant proteins was verified by sequencing PCR fragments amplified from viral DNA.

### Mice

C57BL/6 (B6) were from Fox Chase Cancer Center stock. B6.C-H2bm1/ByJ (bm1, stock #001060) B6.PL-Thy1^a^/CyJ (B6-Thy1.1, stock # 000406), B6.129S7-Rag1^tm1Mom/J^ and (Rag1 KO stock # 002216) were bred at FCCC from mice purchased from Jackson Laboratories (Bar Harbor, Maine). H-2Kb^tm1^, H-2Db^tm1^ (MHC I KO, stock # 004215-MM) were purchased from the Emerging Models Program at Taconic Farms (Germantown, NY) and bred at FCCC. OT-I mice [29], originally a gift from Dr. Stephen Jameson (University of Minnesota, MN), were bred with Rag1 KO and B6-Thy1.1 to homozygosity at FCCC. Bone marrow chimeras were prepared as previously described [6],[7] using 5–7 weeks old mice as donors and recipients. Except for bone marrow chimeras, all experiments used mice between 6–12 weeks of age. Mice were infected or injected with infected cells as indicated. Bone marrow chimeras were prepared as previously described [6],[7]. For CpG treatment, mice were injected intravenously in the tail vein with 20 nM synthetic phosphorothioated CpG1668 (Integrated DNA Technologies Inc, Coralville, IA) [11].

### *In vitro and In vivo* antigen presentation

*In vitro* DP and *in vitro* and *in vivo* CP assays were performed as previously described [16],[42] except that for Ag expression we used recombinant viruses rather than plasmid transfection and infection with WT virus. Thus, for *in vivo* and *in vitro* CP, the virus was inactivated by UV irradiating the Ag donor cells as described [42] and fixing with 2% paraformaldehyde overnight followed by extensive washing. To determine DP by inoculated cells, DC or MC57G cells were infected with VACV, 10 PFU/cell for 1 h, thoroughly washed, and 10^6^ were inoculated into mice as indicated.

### Detection of T cell responses

Determination of proliferation and expansion of CFSE labeled OT-I cells was as before [26]. IIS was performed as previously described [3],[4],[51] except that in some cases, instead of infected cells, the virus-specific T_CD8+_ were restimulated with cells pulsed in complete media with 1 µM synthetic peptides (Genscript corp) for 1 h in CRPMI and thoroughly washed. K^b^-tetramers were produced and used exactly as described [52] except that the SIINFEKL or TSYKFESV peptide were used for the refolding reaction.

### Statistical analyses

One- or two-tailed T test analyses were used according to the hypothesis being tested. Tests were performed using the Graph Pad Prism software.

## Supporting Information

Figure S1 Direct presentation can prime anti-VACV T_CD8+_. A) bm1 mice were adoptively transferred with 10^6^ CFSE labeled OT-I cells and infected IP with 10^6^ PFU of the indicated viruses. OT-I proliferation was determined by FACS on day 4 PI. Data correspond to a pool of three mice and is representative of three experiments. B) bm1 mice were infected with 10^5^ PFU of the indicated viruses IP or SC. Seven days later the SIINFEKL and TSYKFESV-specific T_CD8+_ were determined in the indicated organs by staining with the indicated K^b^ tetramers. Data correspond to two individual mice from groups of two and is representative of three similar experiments. Data is gated on CD8^+^ cells. C) bm1 were infected IP with 10^6^ PFU of the indicated viruses and surface staining with CD8 and the indicates tetramers was performed in splenocytes on day 7 PI. Plots are from a representative mouse and gated on CD8^+^ cells. The graph on the right is the summary for three mice in each group. Gray columns, stained with K^b^-TSYKFESV tetramers; white columns, stained with K^b^-SIINFEKL tetramers. Columns represent the average ±SD of three mice. P values from one-tailed T tests. D) Mice were infected SC with 10^6^ of the indicated viruses and seven days later the SIINFEKL and TSYKFESV-specific T_CD8+_ were determined in the indicated organs by staining with K^b^-SIINFEKL and K^b^-TSYKFESV tetramers. Graphs show the ratio of K^b^-TSYKFESV^+^/K^b^-SIINFEKL^+^ staining for three mice/group. No significant differences between viruses were found by two-tailed T test analysis.
(1.13 MB PDF)

## References

[1] Lau LL, Jamieson BD, Somasundaram T, Ahmed R (1994). Cytotoxic T-cell memory without antigen.. Nature.

[2] Welsh RM, Selin LK, Szomolanyi-Tsuda E (2004). Immunological memory to viral infections.. Annu Rev Immunol.

[3] Fang M, Sigal LJ (2005). Antibodies and CD8+ T Cells Are Complementary and Essential for Natural Resistance to a Highly Lethal Cytopathic Virus.. J Immunol.

[4] Xu R-H, Fang M, Klein-Szanto A, Sigal LJ (2007). Memory CD8+ T cells are gatekeepers of the lymph node draining the site of viral infection.. Proc Natl Acad Sci U S A.

[5] Lenz LL, Butz EA, Bevan MJ (2000). Requirements for bone marrow-derived antigen-presenting cells in priming cytotoxic T cell responses to intracellular pathogens.. J Exp Med.

[6] Sigal LJ, Crotty S, Andino R, Rock KL (1999). Cytotoxic T-cell immunity to virus-infected non-haematopoietic cells requires presentation of exogenous antigen.. Nature.

[7] Sigal LJ, Rock KL (2000). Bone marrow-derived antigen-presenting cells are required for the generation of cytotoxic T lymphocyte responses to viruses and use transporter associated with antigen presentation (TAP)-dependent and - independent pathways of antigen presentation.. J Exp Med.

[8] Heath WR, Belz GT, Behrens GM, Smith CM, Forehan SP (2004). Cross-presentation, dendritic cell subsets, and the generation of immunity to cellular antigens.. Immunol Rev.

[9] Rock KL (1996). A new foreign policy: MHC class I molecules monitor the outside world.. Immunology Today.

[10] Melief CJ (2003). Mini-review: Regulation of cytotoxic T lymphocyte responses by dendritic cells: peaceful coexistence of cross-priming and direct priming?. Eur J Immunol.

[11] Wilson NS, Behrens GM, Lundie RJ, Smith CM, Waithman J (2006). Systemic activation of dendritic cells by Toll-like receptor ligands or malaria infection impairs cross-presentation and antiviral immunity.. Nat Immunol.

[12] Zinkernagel RM (2002). On cross-priming of MHC class I-specific CTL: rule or exception?. Eur J Immunol.

[13] Amigorena S (2003). Y in X priming.. Nat Immunol.

[14] Lizee G, Basha G, Tiong J, Julien JP, Tian M (2003). Control of dendritic cell cross-presentation by the major histocompatibility complex class I cytoplasmic domain.. Nat Immunol.

[15] Heath WR, Carbone FR (2001). Cross-presentation in viral immunity and self-tolerance.. Nat Rev Immunol.

[16] Serna A, Ramirez MC, Soukhanova A, Sigal LJ (2003). Cutting Edge: Efficient MHC Class I Cross-Presentation during Early Vaccinia Infection Requires the Transfer of Proteasomal Intermediates between Antigen Donor and Presenting Cells.. J Immunol.

[17] Norbury CC, Basta S, Donohue KB, Tscharke DC, Princiotta MF (2004). CD8+ T cell cross-priming via transfer of proteasome substrates.. Science.

[18] Restifo NP, Bacik I, Irvine KR, Yewdell JW, McCabe BJ (1995). Antigen processing in vivo and the elicitation of primary CTL responses.. J Immunol.

[19] Wherry EJ, Puorro KA, Porgador A, Eisenlohr LC (1999). The induction of virus-specific CTL as a function of increasing epitope expression: responses rise steadily until excessively high levels of epitope are attained.. J Immunol.

[20] Porgador A, Yewdell JW, Deng Y, Bennink JR, Germain RN (1997). Localization, quantitation, and in situ detection of specific peptide- MHC class I complexes using a monoclonal antibody.. Immunity.

[21] Princiotta MF, Finzi D, Qian SB, Gibbs J, Schuchmann S (2003). Quantitating protein synthesis, degradation, and endogenous antigen processing.. Immunity.

[22] Gilbert PA, McFadden G (2006). Poxvirus cancer therapy.. Recent Pat Antiinfect Drug Discov.

[23] Gherardi MM, Esteban M (2005). Recombinant poxviruses as mucosal vaccine vectors.. J Gen Virol.

[24] Norbury CC, Malide D, Gibbs JS, Bennink JR, Yewdell JW (2002). Visualizing priming of virus-specific CD8+ T cells by infected dendritic cells in vivo.. Nat Immunol.

[25] Hickman HD, Takeda K, Skon CN, Murray FR, Hensley SE (2008). Direct priming of antiviral CD8+ T cells in the peripheral interfollicular region of lymph nodes.. Nat Immunol.

[26] Ma X, Serna A, Xu RH, Sigal LJ (2009). The amino acid sequences flanking an antigenic determinant can strongly affect MHC class I cross-presentation without altering direct presentation.. J Immunol.

[27] Karttunen J, Sanderson S, Shastri N (1992). Detection of rare antigen-presenting cells by the lacZ T-cell activation assay suggests an expression cloning strategy for T-cell antigens.. Proc Natl Acad Sci U S A.

[28] Sanderson S, Shastri N (1994). LacZ inducible, antigen/MHC-specific T cell hybrids.. Int Immunol.

[29] Hogquist KA, Jameson SC, Heath WR, Howard JL, Bevan MJ (1994). T cell receptor antagonist peptides induce positive selection.. Cell.

[30] Clarke SR, Barnden M, Kurts C, Carbone FR, Miller JF (2000). Characterization of the ovalbumin-specific TCR transgenic line OT-I: MHC elements for positive and negative selection.. Immunol Cell Biol.

[31] Kurts C, Kosaka H, Carbone FR, Miller JF, Heath WR (1997). Class I-restricted cross-presentation of exogenous self-antigens leads to deletion of autoreactive CD8(+) T cells.. J Exp Med.

[32] Nikolic-Zugic J, Bevan MJ (1990). Role of self-peptides in positively selecting the T-cell repertoire.. Nature.

[33] Badovinac VP, Haring JS, Harty JT (2007). Initial T cell receptor transgenic cell precursor frequency dictates critical aspects of the CD8(+) T cell response to infection.. Immunity.

[34] Tscharke DC, Karupiah G, Zhou J, Palmore T, Irvine KR (2005). Identification of poxvirus CD8+ T cell determinants to enable rational design and characterization of smallpox vaccines.. J Exp Med.

[35] Qu C, Nguyen VA, Merad M, Randolph GJ (2009). MHC class I/peptide transfer between dendritic cells overcomes poor cross-presentation by monocyte-derived APCs that engulf dying cells.. J Immunol.

[36] Dolan BP, Gibbs KD, Ostrand-Rosenberg S (2006). Dendritic cells cross-dressed with peptide MHC class I complexes prime CD8+ T cells.. J Immunol.

[37] Sollid LM, Vaage JT (2006). Cross-dressing T cells go wild.. Nat Med.

[38] Smyth LA, Herrera OB, Golshayan D, Lombardi G, Lechler RI (2006). A novel pathway of antigen presentation by dendritic and endothelial cells: Implications for allorecognition and infectious diseases.. Transplantation.

[39] Smyth LA, Harker N, Turnbull W, El-Doueik H, Klavinskis L (2008). The relative efficiency of acquisition of MHC:peptide complexes and cross-presentation depends on dendritic cell type.. J Immunol.

[40] Shen L, Sigal LJ, Boes M, Rock KL (2004). Important role of cathepsin S in generating peptides for TAP-independent MHC class I crosspresentation in vivo.. Immunity.

[41] Larsson M, Fonteneau JF, Somersan S, Sanders C, Bickham K (2001). Efficiency of cross presentation of vaccinia virus-derived antigens by human dendritic cells.. Eur J Immunol.

[42] Ramirez MC, Sigal LJ (2002). Macrophages and Dendritic Cells Use the Cytosolic Pathway to Rapidly Cross-Present Antigen from Live, Vaccinia-Infected Cells.. J Immunol.

[43] Gasteiger G, Kastenmuller W, Ljapoci R, Sutter G, Drexler I (2007). Cross-priming of cytotoxic T cells dictates antigen requisites for modified vaccinia virus Ankara vector vaccines.. J Virol.

[44] Basta S, Chen W, Bennink JR, Yewdell JW (2002). Inhibitory effects of cytomegalovirus proteins US2 and US11 point to contributions from direct priming and cross-priming in induction of vaccinia virus-specific CD8(+) T cells.. J Immunol.

[45] Shen X, Wong SB, Buck CB, Zhang J, Siliciano RF (2002). Direct priming and cross-priming contribute differentially to the induction of CD8+ CTL following exposure to vaccinia virus via different routes.. J Immunol.

[46] Ackerman AL, Giodini A, Cresswell P (2006). A role for the endoplasmic reticulum protein retrotranslocation machinery during crosspresentation by dendritic cells.. Immunity.

[47] Junt T, Moseman EA, Iannacone M, Massberg S, Lang PA (2007). Subcapsular sinus macrophages in lymph nodes clear lymph-borne viruses and present them to antiviral B cells.. Nature.

[48] Shastri N, Gonzalez F (1993). Endogenous generation and presentation of the ovalbumin peptide/Kb complex to T cells.. J Immunol.

[49] Earl PL, Cooper N, Wyatt LS, Moss B, Carroll MW (2001). Preparation of cell cultures and vaccinia virus stocks.. Curr Protoc Mol Biol.

[50] Earl PL, Moss B, Wyatt LS, Carroll MW (2001). Generation of recombinant vaccinia viruses.. Curr Protoc Mol Biol.

[51] Fang M, Sigal LJ (2006). Direct CD28 Costimulation Is Required for CD8+ T Cell-Mediated Resistance to an Acute Viral Disease in a Natural Host.. J Immunol.

[52] Rodenko B, Toebes M, Hadrup SR, van Esch WJ, Molenaar AM (2006). Generation of peptide-MHC class I complexes through UV-mediated ligand exchange.. Nat Protoc.

