# Supplementary material for: Direct Presentation Is Sufficient for an Efficient Anti-Viral CD8+ T Cell Response
Source: PLoS Pathog. 2010 Feb 12;6(2):e1000768. doi: 10.1371/journal.ppat.1000768 (PMC2820535; doi:10.1371/journal.ppat.1000768)
Supplement: Figure S1 — Direct presentation can prime anti-VACV TCD8+. A) bm1 mice were adoptively transferred with 106 CFSE labeled OT-I cells and infected IP with 106 PFU of the indicated viruses. OT-I proliferation was determined by FACS on day 4 PI. Data correspond to a pool of three mice and is representative of three experiments. B) bm1 mice were infected with 105 PFU of the indicated viruses IP or SC. Seven days later the SIINFEKL and TSYKFESV-specific TCD8+ were determined in the indicated organs by staining with the indicated Kb tetramers. Data correspond to two individual mice from groups of two and is representative of three similar experiments. Data is gated on CD8+ cells. C) bm1 were infected IP with 106 PFU of the indicated viruses and surface staining with CD8 and the indicates tetramers was performed in splenocytes on day 7 PI. Plots are from a representative mouse and gated on CD8+ cells. The graph on the right is the summary for three mice in each group. Gray columns, stained with Kb-TSYKFESV tetramers; white columns, stained with Kb-SIINFEKL tetramers. Columns represent the average ±SD of three mice. P values from one-tailed T tests. D) Mice were infected SC with 106 of the indicated viruses and seven days later the SIINFEKL and TSYKFESV-specific TCD8+ were determined in the indicated organs by staining with Kb-SIINFEKL and Kb-TSYKFESV tetramers. Graphs show the ratio of Kb-TSYKFESV+/Kb-SIINFEKL+ staining for three mice/group. No significant differences between viruses were found by two-tailed T test analysis. (1.13 MB PDF) [file ppat.1000768.s001.pdf]

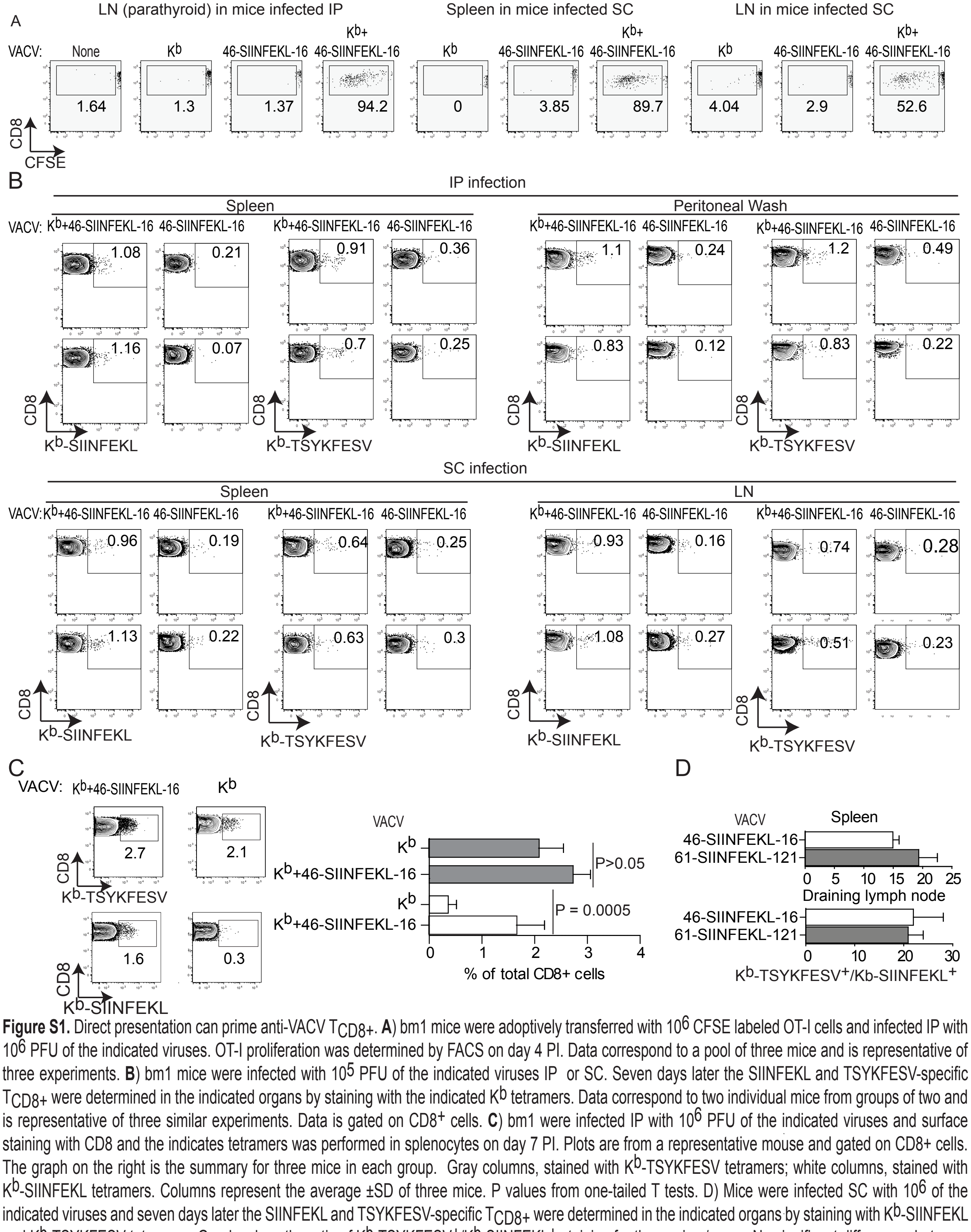

Figure S1. Direct presentation can prime anti-VACV T_{CD8+}. **A**) bm1 mice were adoptively transferred with 10^6 CFSE labeled OT-I cells and infected IP with 10^6 PFU of the indicated viruses. OT-I proliferation was determined by FACS on day 4 PI. Data correspond to a pool of three mice and is representative of three experiments. **B**) bm1 mice were infected with 10^5 PFU of the indicated viruses IP or SC. Seven days later the SIINFEKL and TSYKFESV-specific T_{CD8+} were determined in the indicated organs by staining with the indicated K^b tetramers. Data correspond to two individual mice from groups of two and is representative of three similar experiments. Data is gated on $CD8^+$ cells. **C**) bm1 were infected IP with 10^6 PFU of the indicated viruses and surface staining with CD8 and the indicates tetramers was performed in splenocytes on day 7 PI. Plots are from a representative mouse and gated on CD8+ cells. The graph on the right is the summary for three mice in each group. Gray columns, stained with K^b-TSYKFESV tetramers; white columns, stained with K^b-SIINFEKL tetramers. Columns represent the average ±SD of three mice. P values from one-tailed T tests. D) Mice were infected SC with 10^6 of the indicated viruses and seven days later the SIINFEKL and TSYKFESV-specific T_{CD8+} were determined in the indicated organs by staining with K^b-SIINFEKL and K^b-TSYKFESV tetramers. Graphs show the ratio of K^b-TSYKFESV$^+$/K^b-SIINFEKL$^+$ staining for three mice /group. No significant differences between viruses were found by two-tailed T test analysis.
